# Coordination of adaptive working memory and reinforcement learning systems explaining choice and reaction time in a human experiment

**DOI:** 10.1186/1471-2202-15-S1-P156

**Published:** 2014-07-21

**Authors:** Guillaume D Viejo, Mehdi Khamassi, Andrea Brovelli, Benoît Girard

**Affiliations:** 1 Sorbonne Universités, UPMC, Univ Paris 06, UMR 7222, ISIR, F-75005, Paris, France; 2 CNRS, UMR 7222, ISIR, F-75005, Paris, France; 3 Institut de Neurosciences de la Timone (INT), UMR 7289, CNRS - Aix Marseille Université, Marseille, France

## 

Contemporary behavioral learning theory provides a comprehensive description of how we and other animals learn, and places behavioral flexibility and automaticity at heart of adaptive behaviors. However, to our knowledge, the computations supporting the interactions between *deliberative* and *habitual* decision-making systems are still poorly understood. Previous functional magnetic resonance imaging (fMRI) results suggest that the dorsal striatum host complementary computations that may differentially support *deliberative* and *habitual* processes [1] in the form of a dynamical interplay rather than a serial recruitment of strategies. From the same instrumental task, we develop a dual-system computational model of the two systems that can predict both performance (i.e., participant choices) and modulations in reaction times during learning. The instrumental task is a trial-and-error learning task requiring participants to find the correct associations between color stimuli and finger responses.

To model the habitual system, we use a simple Q-learning algorithm (QL) [2] whose properties are fast responses, but slow convergence. For the deliberative (i.e goal-directed) system, we propose a new Bayesian Working Memory (BWM) which searches for information in the history of previous trials and stops as soon as the uncertainty on the action to perform decreases below a certain threshold. Last, we also propose a model for QL and BWM coordination. Currently, most models of system selection tend to control action selection concurrently, using either the deliberative or habitual model according to uncertainty criteria [3,4]. Only one model has investigated the relation between working memory and reinforcement learning [5] without, however explicitly modeling the temporal aspect of memory manipulation. In our approach, we propose a model for QL and BWM coordination. QL and BWM are merged such that the expensive memory manipulation is under control of, among others, the level of convergence of the habitual learning. Consequently, we also predict specific reaction times for each model that can be compared with the evolution of reaction times in instrumental learning tasks.

Models are optimized for each subject with the NSGA-2 multi-objective evolutionary algorithm. The first fitness function is the Bayesian Information Criterion for individual choices. The second fitness function is also a likelihood that maximizes the probability of performing reaction times similar to humans. We compare the ability of the new model to explain human behavior with the QL or BWM only, as well as with a combination of these models based on [4], which reveals that the proposed model is in general more accurate. To conclude, we suggest that a close combination of BWM and QL better explains both choices and reaction times for most participants.
